# Use of electronic medical records and quality of patient data: different reaction patterns of doctors and nurses to the hospital organization

**DOI:** 10.1186/s12911-017-0412-x

**Published:** 2017-02-10

**Authors:** Mattijs S. Lambooij, Hanneke W. Drewes, Ferry Koster

**Affiliations:** 1 Department Quality of care and health Economics, National Institute for Public Health and the Environment (RIVM), PO Box 1, 3720 BA Bilthoven, The Netherlands; 2 Department Quality of Care and Health Economics, National Institute for Public Health and the Environment (RIVM), Center for Nutrition, Prevention and Health Services, PO Box 1, 3720 BA Bilthoven, The Netherlands; 3 Department of Sociology, Erasmus University Rotterdam, Rotterdam and TIAS School for Business and Society, Tilburg, The Netherlands

**Keywords:** Electronic medical records, Health personnel, Hospital, Health services, Implementation process, Quality of patient data

## Abstract

**Background:**

As the implementation of Electronic Medical Records (EMRs) in hospitals may be challenged by different responses of different user groups, this paper examines the differences between doctors and nurses in their response to the implementation and use of EMRs in their hospital and how this affects the perceived quality of the data in EMRs.

**Methods:**

Questionnaire data of 402 doctors and 512 nurses who had experience with the implementation and the use of EMRs in hospitals was analysed with Multi group Structural equation modelling (SEM). The models included measures of organisational factors, results of the implementation (ease of use and alignment of EMR with daily routine), perceived added value, timeliness of use and perceived quality of patient data.

**Results:**

Doctors and nurses differ in their response to the organisational factors (support of IT, HR and administrative departments) considering the success of the implementation. Nurses respond to culture while doctors do not. Doctors and nurses agree that an EMR that is easier to work with and better aligned with their work has more added value, but for the doctors this is more pronounced. The doctors and nurses perceive that the quality of the patient data is better when EMRs are easier to use and better aligned with their daily routine.

**Conclusions:**

The result of the implementation, in terms of ease of use and alignment with work, seems to affect the perceived quality of patient data more strongly than timeliness of entering patient data. Doctors and nurses value bottom-up communication and support of the IT department for the result of the implementation, and nurses respond to an open and innovative organisational culture.

**Electronic supplementary material:**

The online version of this article (doi:10.1186/s12911-017-0412-x) contains supplementary material, which is available to authorized users.

## Background

Electronic Medical Records (EMRs) are widely implemented in health care organisations, and much is known about factors affecting implementation processes. Despite the expected benefits of EMRs [1–3] the adoption of EMRs proceeds slowly [3, 4]. The body of literature of implementation of EMRs is considerable and many competing theories and models exist. In many of those theories and models, the organisation, the type of innovation or technology and the user [1, 5–9] play a role. Evidence on organisational factors affecting innovation processes in health care organisations is mixed [10]. Some studies find positive associations [11] and others find little or no effect on the innovation process [12, 13]. Reasons for users to resist the implementation of an EMR are a poor design [14], too much pressure to adopt the EMR [15] or a lack of user involvement in the implementation process [16, 17].

For implementation processes to be successful and to prevent adverse effects it is essential that one considers the interaction between all user groups, the organisation and the innovation. Taking account of potential differences in responses between the user groups increases the chance of a successful implementation of EMRs [18, 19].

We tried to construct a model that was based on knowledge in the literature and searched for operationalisations used in similar research. We build on the knowledge that the environment in which the innovation is adopted is essential for realisation of its potential [20]. An assumption in this paper is that a successful implementation of an EMR improves the quality of patient data. A new element of our study is that we investigate differences in responses to the EMRs between two user groups, doctors and nurses.

Aspects such as organisational culture and climate, leadership style, power balance and social relations [21] have been found to affect the uptake of innovations. When implementing an IT innovation the “soft” social and cultural aspects are as important as the “hard” technical and product specifications [22, 23]. Adding to the complexity is the fact that EMRs are used by different groups that need to work as a team and at the same time have different positions in the hospitals and can therefore be expected to react differently to organisational factors [24–27]. Implementation processes require users to change their work routines and they may be confronted with disadvantages including loss of autonomy [24, 28, 29]. Advantages and disadvantages may differ between user groups; their expectations may differ [30], their preferences may differ [31] and they may prefer different ways to use EMRs [32]. Taking account of the knowledge in the literature, and taking account of possible different reactions between user groups, we constructed a model that includes paths that lead from organisational factors to the outcome of the implementation process and the subsequent added value for its users in their daily routine [7], which ultimately affects the quality of patient data in the EMR. We hypothesise that the quality of the IT innovation is better if the quality of the organisational system is better and that this positively affect satisfaction of the user, EMR use and quality of patient data. This will affect both the individual and the organisational impact of the newly implemented system. We also expect that organisational factors are more important with regard to the successful implementation and use for nurses than for doctors. The main reason being that most medical specialists work in partnerships within hospitals, while nurses are employed by hospitals. The research question of this paper is: Which organisational factors affect the implementation success of the EMR as perceived by the user groups doctors and nurses most strongly and (on which aspects) does this differ between doctors and nurses?

## Methods

### Questionnaire and data collection

Doctors and nurses were invited to complete an on-line questionnaire if they worked in hospitals where an EMR was being implemented at the time of the data collection. The questionnaire contained items measuring the constructs of the theoretical model. The items in the questionnaire were based on previous research as much as possible. When needed, new items were developed (Additional file 1 presents the items). The constructs and links to theories are presented in the “Study variables” section.

The questionnaire was designed specifically for this study and used one time. The doctors and nurses were recruited from two separate research voluntary registries. The registry of the doctors contains physicians who volunteered to cooperate with studies by answering questionnaires. These volunteers were invited to participate in this study, explaining that is was a study on EMR use. The nurses were selected from a general research voluntary registry. In this registry nurses were selected based on their reported occupation. These nurses were invited to reply to the questionnaire similarly to the doctors. First, they were asked whether they currently worked with EMRs in their hospitals. Only those nurses who scored a “yes” on this question could participate in the present study. Both questionnaires were online for about two weeks. The respondents received a reward after completing the questionnaire,. Doctors received a monetary reward and the nurses were given “points” that enabled them to buy products. No reminders were sent.

### Analyses

The data were analysed using Structural Equation Modelling (SEM), which combines path-analysis, simultaneous-equation models and factor analysis [33, 34]. It enables estimating associations of latent variables (unobserved variables, measured with multiple items) and estimating multiple regression equations, including more than one dependent variable in one model. The results show the level of fit between the observed items and the latent variables. Regression parameters express the strength of the association between the latent variables. The evaluation of model fit is presented using three fit measures [35]: the RMSEA (Root Mean Square Error of Approximation), the CFI (Comparative Fit Index), and the TLI (Tucker Lewis Index). Each measure has to meet a particular threshold to indicate a good fit. We used the package ‘lavaan’ in R to conduct the analyses [33].

### Study variables

**Fig. 1:**
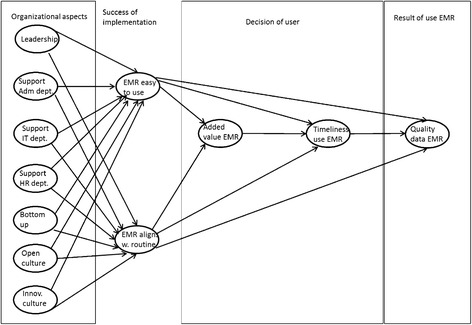
Conceptual model (all expected signs of the paths are positive (+)). Leadership = authentic leadership; Support Adm dept = support of administrative department; Support IT dept. = Support of IT department; Support of HR dept = Support of HR department; Bottom-up = Bottom-up communication in the hospital; Cultopen = Open culture in hospital; Innov culture = Innovative culture in hospital; EMR easy to use = EMR is easy to work with; EMR aligns w. routine = Alignment of EMR to routine; Added value EMR = added value of EMR use by users; Timeliness use EMR = Timeliness of entering patient data in EMR, reported by users; Quality data EMR = Quality of patient data in EMR)

The model that we built to test our hypotheses consists of four parts: Organisational aspects, Success of implementation, Decision of user and Result of using the EMR (Fig. 1 presents the model).

#### Organisational aspects

The social system that needs to adopt the innovation [8] is the hospital and the hospital contains a number of elements affecting the implementation process [36, 37]. There are several management tools and both “hard” organisational aspects and “soft” cultural and social aspects have been found to influence innovation [6, 21, 22, 37, 38]. In the following sections, we present the elements of the empirical model and literature of implementation research related to the subsequent elements.

##### Management tools

The management of a hospital has several tools to guide organisational processes. Authentic leadership (Fig. 1) tends to affect other organisation members (e.g. clinicians) in that they legitimise management’s role to guide the organisation [21, 39–41] and that they more readily accept organisational changes. Bottom-up influence is related to leadership and is known to affect the support of EMRs [16, 17, 41–43]. The model includes these two management tools. The precise wordings of the items are presented in the Additional file 1. Authentic leadership has a Cronbach’s alpha of 0.92 and bottom-up communication has a Cronbach’s alpha of 0.90.

##### Support from other departments

An other organisational aspect that we included in the model is support from other departments. Starting to work with an EMR requires new skills that need specific education and training during the implementation stage [44]. The HR department may help identifying these needs and offer targeted courses and training [21], thus improving the performance of the clinicians working with the EMR [45]. IT support may help to overcome practical problems. Hence, support from the IT department may play an important role during and after the implementation stage [46]. And because of the intrinsic link between EMR and administration, having administrative staff that supports users with entering patient data is likely to affect the successful implementation and use of an EMR [19]. The model includes three constructs for support from the IT, HR and administrative department. See the Additional file 1 for precise wordings. The Cronbach’s alphas of the scales are 0.91 (IT department.), 0.90 (HR department) and 0.93 (administrative department).

##### Organisational culture

A culture that enhances communication [47], or a culture that is innovative [48] and open [21, 42], is likely to contribute to the success of the implementation of EMR. We included two measures of culture: open culture and innovative culture, based on work of Woerkom [49] and de Jong et al. [50] respectively. The Cronbach’s alphas are 0.70 for innovative culture and 0.76 for open culture.

#### Success of implementation

The success of implementation is measured with two items: ease of use and alignment with daily routine [51]. This notion is based on the Technology Acceptance Model (e.g. [52]). The result of the EMR implementation is measured with two variables: ease of use and alignment with daily routine of the user groups [51]. The Cronbach’s alphas are 0.87 and 0.92 respectively.

#### Adoption decision by user

When the added value of good quality patient data becomes clear to the user, the satisfaction with the system as well as its use are likely to increase [53]. We assume that the fit between technology, user and task affects the use of the innovation [5] in the sense that a better fit between daily routine and ease of use is likely to further the use of the innovation. We expect that ease of use and alignment of EMR to the daily routine will positively affect the perceived added value of EMR and that the users consequently will enter the patient data more timely in the EMR.

#### Data quality in EMR

The ultimate dependent variable, presented in Fig. 1 in the far right “result of use” section, represents the quality of the data in EMR as reported by its users (cf. organisational impact in [7]). It was assumed that if the data represents reality in terms of the patient’s health and the care provided accurately and completely [54], its information will support the users (i.e. clinicians) in their work. Then the users will take full advantage of the potential of the EMR and it will support the organisation in process management, e.g. accurate billing [25].

Three factors are expected to influence quality of the data. First, by the way in which patient data are entered into the EMR (Fig. 1 section “Decision of user”). We assumed that the quality of the data is higher when the time between seeing the patient and entering the patient data is shorter. Second, when the EMR is easy to use, and this may be the result of a well-functioning implementation process, the quality of the data is higher. Third, a better alignment of the EMR functionalities and the daily routine of its users [14] is also expected to positively affect the quality of the data.

#### Control variables

The control variables are gender (0 = male and 1 = female), age and level of implementation of the EMR at the time of data collection. It is known that the implementation stage of the EMR affects the perception and support of users of EMRs [55, 56]. The measurement of level of implementation is based on answers of respondents about computerisation of EMR in their hospital is (next to using paper files). The score is higher for respondents working with a completely computerised EMR. If the complete EMR was reported to consist of a single system, the score on this variable is higher (+1), and if the EMR consists of multiple systems (and not one integrated system), the score is lower (+0.5). When the data entered by nurses are visible to doctors and vice versa, the score of implementation level is also higher (+1).

### Comparison of user groups

The groups (doctors and nurses) are compared in two ways. First, we investigate whether the doctors and nurses differ in the regression parameters in differing from zero. Second, we compare significant differences of the regression coefficients between the groups by re-estimating the multi group model, but constraining the regression parameters to be similar across groups. Subsequently, we ran models in which the parameter of interest was allowed to differ. Subsequently, the chi^2^ change of the two models is compared. If the chi^2^ improves significantly, we conclude that the doctors and nurses differ in behaviour on this regression parameter. The differences in chi^2^ and subsequent significance levels are reported in the right hand columns of Table 2.

## Results

### Sample

474 doctors (24%) and 699 (19%) nurses returned the questionnaires. Only complete questionnaires were included. Due to item non-response, 914 respondents were included in the analyses (402 doctors and 512 nurses). Where possible, open answers were used to add to the closed questions, e.g. on the question of quality of the data. Sample characteristics are shown in Table 1. 17% of the nurses reported to work in an academic hospital, 22% in a top-clinical hospital, 50% in a general hospital, 3% in a specialized hospital, 7% in a mental hospital, and 1% in a private hospital. In the Netherlands, there are 85 hospitals, 8 of which are academic hospitals (9.4%), and 90.6% are general or specialized hospitals.^1^ This means that in our respondent group, nurses and doctors from academic hospitals are overrepresented. This can be explained by the fact that we intended to include only nurses and doctors who had experience with working with EMR and that Dutch academic hospitals are at the forefront of implementing EMR.

**Table 1:** Sample characteristics

	Nurses (512)	Doctors (402)
Mean age (SD)	42.52 (11.58)	47.6 (9.6)
% female	80%	28%
Reported type of hospital		
Academic	17%	24%
Top clinical	22%	30%
General	50%	41%
Specialized	3%	1%
Private	1%	2%

### Analyses

**Fig. 2:**
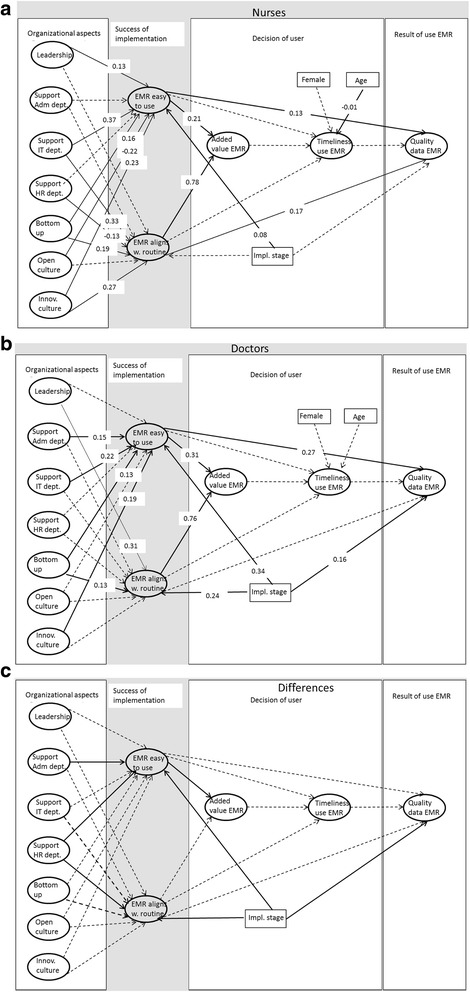
**a** Regression paths SEM model nurses. Leadership = authentic leadership; Support Adm dept = support of administrative department; Support IT dept. = Support of IT department; Support of HR dept = Support of HR department; Bottom-up = Bottom-up communication in the hospital; Cultopen = Open culture in hospital; Innov culture = Innovative culture in hospital; EMR easy to use = EMR is easy to work with; EMR aligns w. routine = Alignment of EMR to daily tasks; Added value EMR = added value of EMR use by users; Timeliness use EMR = Timeliness of entering patient data in EMR, reported by users; Quality data EMR = Quality of patient data in EMR). **b** Regression paths SEM model doctors. **c** Differences in responses between doctors and nurses. The *solid arrow* represent the paths of which the doctors and nurses differ (see table 3 far *right column*), *dotted arrow* represent paths with similar results for doctors and nurses

Table 2 and Fig. 2a,b and c present the main results of the analyses. Table 2 shows the regression coefficients of the multi group model for the two groups (doctors and nurses) and the analyses of the difference between the two groups. In the right columns, the chi^2^ difference with one degree of freedom is presented of a model with constraint regression parameters and a model with that particular regression parameter allowed to differ between the groups. If the difference is significant, this means that the nurses and doctors differ on this parameter. Figure 2a presents the SEM model for nurses and Fig. 2b for doctors. Figure 2c presents the differences between the two groups. The solid arrows represent the significant regression paths and the dotted arrow the non-significant results. The multi group model (no constraints) has a good fit: chi^2^ (1966) = 3852.62; CFI = 0.93; TLI = 0.93; RMSEA = 0.046 (95% CI = 0.044-0.048).

**Table 2:** Regression parameters of multi group SEM model, regression parameter reported separately for nurses, and doctors

		Doctors			Nurses			Difference Doct/Nurse	
Dependent variables	Independent variables	b		s.e.	b		s.e.	Chi^2^ diff (1)	
								Link with Fig. 1	
Easy to use								Organizational aspects and success of implementation	
	Leadership	0.11		0.10	0.13		0.07	0.09	
	Support administrative dept.	0.15		0.09	−0.03		0.07	4.39	*
	Support IT dept.	0.22	*	0.09	0.37	**	0.06	1.20	
	Support HR dept.	0.08		0.05	−0.05		0.04	4.27	*
	Bottom-up options	0.13	*	0.05	0.16	**	0.05	0.23	
	Open culture	−0.12		0.16	−0.22	*	0.11	0.50	
	Innovative culture	0.19		0.11	0.23	*	0.10	1.21	
Alignment with work									
	Leadership	0.31	**	0.11	0.09		0.08	1.70	
	Support administrative dept.	0.07		0.10	−0.03		0.08	0.62	
	Support IT dept.	0.15		0.10	0.33	**	0.07	0.05	
	Support HR dept.	0.00		0.06	−0.13	**	0.05	3.33	
	Bottom-up options	0.13	*	0.06	0.19	**	0.06	0.06	
	Open culture	0.17		0.18	−0.14		0.12	2.09	
	Innovative culture	0.08		0.12	0.27	*	0.11	0.57	
								Decision of user	
Added value									
	Easy to use	0.31	**	0.04	0.21	**	0.04	3.61	
	Alignment with work	0.76	**	0.05	0.78	**	0.05	0.71	
Timeliness of use									
	Added value	0.05		0.11	−0.04		0.16	3.64	
	Easy to use	−0.07		0.06	−0.14		0.08	3.58	
	Alignment with work	−0.08		0.10	0.06		0.15	3.12	
Quality data								Result of EMR use and implementation	
	Timeliness of use	0.02		0.04	−0.03		0.03	0.67	
	Easy to use	0.27	**	0.04	0.13	**	0.04	2.57	
	Alignment with work	0.06		0.04	0.17	**	0.04	0.04	
Quality data									Control variables
	Implementation stage	0.16	**	0.05	0.01		0.04	7.75	**
Alignment with work									
	Implementation stage	0.24	**	0.04	0.05		0.04	6.15	*
Easy to use									
	Implementation stage	0.34	**	0.06	0.08	*	0.04	15.48	**
Timeliness of use									
	Age	−0.01		0.00	−0.01		0.01	0.51	
	Female	0.06		0.08	0.12		0.11	2.16	

** = *p* < 0.01; * = *p* < 0.05

### Organisational factors and result of implementation

Regression coefficients (reported in B and s.e.) and differences between the groups (the chi^2^ of model fit) in Table 2 show that nurses and doctors value the influence of organisational factors on the success of implementation differently. In line with the expectations, the doctors report that their EMR is easier to work with if there is more support from the administrative department (B = 0.15, s.e. = 0.09), more support from the IT department (B = 0.22, s.e. = 0.09), more bottom-up communication (B = 0.13, s.e. = 0.05) and a more innovative culture (B = 0.19, s.e. = 0.11). The nurses find their EMR easier to work with if there is more authentic leadership (B = 0.13, s.e. = 0.07) and more support from the IT department (B = 0.37, s.e. = 0.06) and bottom-up communication (0.16, s.e. = 0.05). The nurses find the EMR easier to work with when the culture is less open (B = −0.21, s.e. = 0.11) and when the culture is more innovative (B = 0.23, s.e. = 0.10). The doctors and nurses differ in the influence of the support of the administrative department (chi^2^ difference = (4.39 (1)) and of the HR department (chi^2^ difference = 4.27 (1)).

Doctors find the EMR to be better aligned with their daily routine if there is more authentic leadership (B = 0.31, s.e. = 0.11) and more bottom-up communication in the hospital (B = 0.13, s.e. = 0.06). The nurses find their EMR to be better aligned with their daily routine when they get more support of the IT department (B = 0.33, s.e. = 0.07), but the HR department is negatively affect the alignment of the EMR (B = −0.13, s.e. = 0.05). In line with our expectations, nurses find the EMR better aligned with their work when there is more bottom-up communication (B = 0.19, s.e. = 0.06) and an innovative culture (B = 0.27, s.e. = 0.11).

### Success of implementation, added for value users and subsequent use

The added value of the users of the EMR increases with easiness to work with and with alignment to their work for both doctors (B = 0.31; s.e. = 0.04 and B = 0.76; s.e. = 0.05 respectively) and nurses (B = 0.21; s.e. = 0.04 and B = 0.78; s.e. = 0.05).

### Quality of patient data

Doctors find the patient data in the EMR of better quality when the EMR is easier to use (B = 0.27, s.e. = 0.04). The nurses also find the quality of the patient data to be better when the EMR is easier to use (B = 0.13, s.e. = 0.04) and nurses report that the quality of the data is better when the alignment to work is better (B = 0.17, s.e. = 0.04). The doctors and nurses do not differ in these aspects.

Concerning the control variables, we see that doctors and nurses react differently to the level of implementation. This affects the nurses less than the doctors in their opinion on how it aligns with daily routine (chi^2^ difference = 6.15 (1)) and quality of the data (chi^2^ difference = 7.75 (1)).

## Discussion

This study aimed to increase our understanding of which organisational factors affect the success of implementation processes and how this may differ between doctors and nurses. We tried to construct a model that was based on knowledge in the literature and searched for operationalisations used in similar research. The body of literature is considerable and many competing theories and models exist. In many theories and models, the organisation, the innovation or technology and the user [1, 5–9] play a role. In many theories and models the purpose of an implementation process, i.e. to improve the status quo, remains more or less implicit, but we tried to include an outcome measure in the model: quality of the patient data. A new element of our study is that we applied the model to two different user groups (doctors and nurses) and found that they responded differently in a number of ways. Differences between stakeholders in implementation processes are acknowledged previously, but as far as we know, this is the first study to show that different user groups react differently to the same organisational aspects in an implementation process.

The most prominent differences between the doctors and the nurses are in their estimations of the influence of organisational support on implementation success. We find that the doctors and nurses differ in reaction to the support of the HR department. While the nurses tend to value HR support negatively, the doctors appear to state that HR has little influence. The results of bottom-up communication are positive as expected for both user groups and when the EMRs are easier to use and better aligned with the work of the users, the added value is perceived to be higher.

We expected to find that clinicians who saw fewer benefits from working with the EMR would be more inclined to delay entering the patient data. However, organisational aspects were found to restrict much of the opportunities of the clinicians to hamper the use of the EMR. The characteristics of the result of the implementation are more closely associated with the use of the EMR. When the EMR is perceived as easy to use and is better aligned with their daily routines, less time elapses between the patient’s visit and entering the patient data. The data indicate that doctors’ opinions change stronger after working with the EMRs than the nurses’, doctors tend to be more positive about how the EMR aligns with their routine and about quality of patient data. We did not find this for the nurses.

In this study, an innovative culture implies that colleagues and managers are inclined to consider new ideas to improve work processes. Interestingly, we found that an open culture, which is open to discussions on suboptimal performance, did not result in a better outcome of the implementation process. Contrary to expectations, nurses who had more opportunities to discuss problems with colleagues and management also found the EMR harder to work with. An explanation might be that in an innovative culture, the employees are encouraged to try new things, and not to dwell on what went wrong due to new working methods.

It should also be noted that the data used to investigate the measurement of the theoretical constructs relies on the perceptions of members of the organisation as they are reported by members of the organisation. This means that we did not investigate an ‘objective’ measure. Although such information is insightful, for example because organisational members have more confidence in projects if they believe that the management of their organisation can manage them appropriately, it should be acknowledged that these perceptions may not fully capture the actual situation in an organisation. This can be seen as a limitation, but may also be argued that the perceived situations (such as culture but also quality of the data) is what really affects the work processes of the doctors and nurses, more than the objective situation. If management for instance reports to ensure sufficient bottom-up communication, but doctors and nurses experience differently, measuring this parameter at the managerial level is not likely to enhance our understanding of the mechanisms that affect the use of innovations. Measurements of the factors in the model may be improved by combined views of more stakeholders and study where views overlap. Measurement of the quality of the data of EMRs can be measured even better since the files are stored and can be retrieved at a later moment in time. Therefore, improved measurements of the quality of data are for instance to retrospectively rate consistency and completeness of the patient files. This study shows that different actors react differently to the same organisational factors and future studies may focus on differences and similarities of perceptions of organisational factors and how these affect implementation processes.

We constructed the models based on the theoretical consideration presented in the introduction. First, we constructed the scales based on factor analyses. We confirmed the results with reliability analyses, to estimate the Cronbach’s alpha of the scales. Next we built the model, assuming that organisational factors would affect the result of the implementation process and that in turn this would affect the response of the users and subsequent quality of use of the innovation. We took all organisational factors as a starting point without hypothesising on possible causation among these factors. It could also be argued for instance that leadership and bottom-up communication are enablers for the quality of practical support of the HR, IT and administrative departments. This could then maybe provide a mechanism to explain the effects of leadership and bottom up communication. However, given that both aspects also appear to have direct relations with the variables that indicate the success of the implementation, we assume that this model is sufficiently close to reality without having to add to the complexity of the model.

Given the relatively low response and the overrepresentation of respondents working in academic hospitals, the analyses may be biased to a certain extent. In general, academic centres are first to adopt novel techniques and people working in academic hospitals may be more inclined to use innovations than doctors and nurses in non-academic hospitals. By implication, in particular the variance in the attitudinal measure is lower than it would have been if the sample were representative on this parameter. Part of the lack of significance of the attitudinal variable may be that the respondents can do little else but to adopt an innovation that is implemented organisation-wide.

## Conclusions

Doctors and nurses differ in a number of aspects in their response to (new) use of EMRs. Bottom-up influence gave the most coherent results: for both doctors and nurses, the success of the innovation came with more bottom-up communication. A second relatively consistent finding is that support of the IT departments yields a positive result on EMR implementation. Doctors and nurses differ in their responses to the support of other department. Organisational culture had some influence but it seems to be less important and may work as a negative factor in use of EMRs.

By comparing the different factors that can influence the success of implementation, this study contributes to the scientific field of innovation and the implementation of new technology. We found indications that characteristics of the EMR (ease of use and alignment with tasks) have more influence on the quality of the data in the EMR than timely entering of patient data by the user groups.

For hospital managers, results of this study are directly applicable. Many of the actions they take, may result in differences in reactions from the user groups, sometimes in subtle but possibly relevant. However, when the users have bottom-up influence during and after the implementation process and when the IT department has the skills and ability to give optimal support to the users, this may positively affect the implementation success. In turn more successful the results of the implementation process, the better the quality of the data in the EMR, regardless of how timely the users enter the data into the system.

## Additional files

Additional file 1: Latent constructs, manifest items, coding and factor loadings. Table with exact wording of questionnaire, factor loadings of items, ordered by scales, including Cronbach’s alpha’s. (DOCX 36 kb)
Additional file 2: Data of SEM model in CSV format. Raw data of included cases. The sex and age were removed on request by the journal for protection of privacy for the respondents. (CSV 102 kb)
Additional file 3: Codebook to the data. Variable names in the data and translated complete wording of items in questionnaire. Names of variables in Additional file 2 and the items in complete wording as presented in the Quesionnaire, translation from Dutch. (DOCX 19 kb)
Additional file 4: Complete Dutch questionnaire. (DOCX 25 kb)

## Abbreviations

CFI: Comparative fit index
EMR: Electronic medical record
HR: Human resources
IT: Information technology
RMSEA: Root mean square error of approximation
SEM: Structural equation modelling
TLI: Tucker lewis index

## Footnotes

1
http://statline.cbs.nl/StatWeb/publication/?DM=SLNL&PA=81451ned&D1=0-2&D2=0-2&D3=a&HDR=G1%2CG2&STB=T&VW=T. Consulted May 8 2015
