# Supplementary material for: Use of electronic medical records and quality of patient data: different reaction patterns of doctors and nurses to the hospital organization
Source: BMC Med Inform Decis Mak. 2017 Feb 10;17:17. doi: 10.1186/s12911-017-0412-x (PMC5303309; doi:10.1186/s12911-017-0412-x)
Supplement: Additional file 1: — Latent constructs, manifest items, coding and factor loadings. Table with exact wording of questionnaire, factor loadings of items, ordered by scales, including Cronbach’s alpha’s. (DOCX 36 kb) [file 12911_2017_412_MOESM1_ESM.docx]

***Additional file 1: Latent constructs, manifest items, coding and factor loadings***

| **Innovative culture**  (Cronbach’s alpha 0.70) |  | | |  | Mean(sd) | Nurses Estimate |  | s.e. | Doctors  Estimate |  | s.e. |
| --- | --- | --- | --- | --- | --- | --- | --- | --- | --- | --- | --- |
|  | epd_27_t1 | | | The hospital encourages me to try out new ideas that may lead to improved work processes | 3.31(0.81) |  |  |  |  |  |  |
|  | | | |  |  |  |  |  |  |  |  |
|  | epd_27_t2 | | | If I try to improve a work process, my colleagues are open to it | 3.60(0.71) | 0.66 | ** | 0.06 | 0.52 | ** | 0.06 |
|  | epd_27_t4 | | | My manager listens to me when I come up with an idea to improve the work process | 3.83(0.83) | 0.81 | ** | 0.07 | 0.31 | ** | 0.04 |
|  |  | | | 1=completely disagree, 5=completely agree |  |  |  |  |  |  |  |
|  |  | | |  |  |  |  |  |  |  |  |
| **Open culture**  (Cronbach’s alpha 0.76)  *Covaries with* | | | |  |  |  |  |  |  |  |  |
| *epd_29_t2* | epd_29_t1 | | | If I feel that I fail to do my job properly, I will discuss this with my team members | 3.62(0.75) | 1.00 |  |  | 1.00 |  |  |
| *epd_29_t1* | epd_29_t2 | | | I regularly ask my team members for advice | 3.55(0.67) | 0.73 | ** | 0.08 | 0.89 | ** | 0.13 |
|  | epd_29_t3 | | | If I feel that I fail to do my job properly, I will discuss this with my manager | 3.06(0.89) | 2.15 | ** | 0.21 | 1.76 | ** | 0.30 |
|  | epd_29_t4 | | | I regularly ask my manager for advice | 2.83(0.88) | 1.70 | ** | 0.16 | 1.65 | ** | 0.27 |
|  |  | | | 1=completely disagree, 5=completely agree |  |  |  |  |  |  |  |
| **IT support**  (Cronbach’s alpha 0.91) | | | | The IT department….. |  |  |  |  |  |  |  |
|  | epd_23_t1 | | | reacts swiftly and adequately to problems with registration in the EMR | 3.43(1.09) | 1.00 |  |  | 1.00 |  |  |
|  | epd_23_t2 | | | has the necessary skills and expertise to support proper functioning of the EMR | 3.53(1.03) | 1.05 | ** | 0.04 | 1.21 | ** | 0.07 |
|  | epd_23_t3 | | | has the necessary skills and expertise to align the EMR to my work | 3.35(1.08) | 1.06 | ** | 0.04 | 1.26 | ** | 0.07 |
|  |  | | | 1=completely disagree, 5=completely agree |  |  |  |  |  |  |  |
| **HR support**  (Cronbach’s alpha 0.90) | | | | The HR department….. |  |  |  |  |  |  |  |
|  | epd_22_t1 | | | reacts swiftly and adequately to problems with registration in the EMR | 3.36(1.06) | 1.00 |  |  | 1.00 |  |  |
|  | epd_22_t2 | | | has the necessary skills and expertise to support proper functioning of the EMR | 3.22(1.01) | 1.08 | ** | 0.04 | 1.08 | ** | 0.05 |
|  | epd_22_t3 | | | has the necessary skills and expertise to align the EMR to my work | 3.06(1.03) | 0.99 | ** | 0.04 | 0.97 | ** | 0.05 |
|  |  | | | 1=completely disagree, 5=completely agree |  |  |  |  |  |  |  |
| **Administrative support**  (Cronbach’s alpha 0.93) | | | | The administrative department….. |  |  |  |  |  |  |  |
|  | epd_24_t1 | | | reacts swiftly and adequately to problems with registration in the EMR | 3.39(0.93) | 1.00 |  |  | 1.00 |  |  |
|  | epd_24_t2 | | | has the necessary skills and expertise to support proper functioning of the EMR | 3.40(0.89) | 1.09 | ** | 0.04 | 1.12 | ** | 0.05 |
|  | epd_24_t3 | | | has the necessary skills and expertise to align the EMR to my work | 3.31(0.93) | 1.12 | ** | 0.04 | 1.17 | ** | 0.06 |
|  |  | | | 1=completely disagree, 5=completely agree |  |  |  |  |  |  |  |
|  |  | **Leadership management authentic**  (Cronbach’s alpha 0.92)  Management of this hospital….. | | | | |  |  |  |  |  |
|  | epd_18_t1 | | | supports staff to improve interaction with others within the hospital | 3.27(0.95) | 1.00 |  |  | 1.00 |  |  |
|  | epd_18_t2 | | | analyses relevant information before making decisions | 3.16(0.93) | 0.93 | ** | 0.05 | 1.14 | ** | 0.08 |
|  | epd_18_t3 | | | lets me know how they judge my skills | 2.82(1.01) | 0.98 | ** | 0.06 | 0.85 | ** | 0.08 |
|  | epd_18_t4 | | | admits mistakes | 2.91(0.93) | 0.96 | ** | 0.05 | 1.08 | ** | 0.08 |
|  | epd_18_t5 | | | listens carefully to different points of view before drawing conclusions | 2.99(0.92) | 1.10 | ** | 0.05 | 1.25 | ** | 0.08 |
|  | epd_18_t6 | | | knows when to reconsider its points of view | 2.97(0.88) | 1.00 | ** | 0.05 | 1.16 | ** | 0.07 |
|  | epd_18_t7 | | | encourages everybody to express his or her opinion | 3.24(0.96) | 1.03 | ** | 0.06 | 1.06 | ** | 0.08 |
|  | epd_18_t8 | | | supports staff | 3.29(0.91) | 1.00 | ** | 0.05 | 1.08 | ** | 0.07 |
|  |  | | | 1=completely disagree, 5=completely agree |  |  |  |  |  |  |  |
|  |  | **Bottom-up implementation**  (Cronbach’s alpha 0.90)  *Covaries with* | | | | |  |  |  |  |  |
| *epd_21_t2* | epd_21_t1 | | | I was asked how the EMR should be implemented beforehand | 2.49(1.21) | 1.00 |  |  | 1.00 |  |  |
| *epd_21_t1* | epd_21_t2 | | | Prior to the implementation, I was asked what I needed to improve my performance | 2.64(1.18) | 1.07 | ** | 0.04 | 0.98 | ** | 0.05 |
| *epd_21_t4* | epd_21_t3 | | | During implementation I could tell what I thought of the implementation | 2.86(1.14) | 1.08 | ** | 0.07 | 0.88 | ** | 0.08 |
| *epd_21_t3* | epd_21_t4 | | | During implementation I could tell what I wanted differently in the EMR | 2.86(1.14) | 1.05 | ** | 0.07 | 0.81 | ** | 0.08 |
|  |  | | | 1=completely disagree, 5=completely agree |  |  |  |  |  |  |  |
|  |  |  |  |  |  |  |  |  |  |  |  |
| **EMR easy to work with** (Cronbach’s alpha 0.87) | | | |  |  |  |  |  |  |  |  |
|  | epd_16_t1 | | | Working with the EMR is clear and easy to understand | 3.55(0.98) | 1.00 |  |  | 1.00 |  |  |
|  | epd_16_t2 | | | It is easy for me to make the EMR do what I want | 3.01(1.05) | 1.04 | ** | 0.05 | 0.99 | ** | 0.05 |
|  | epd_16_t3 | | | The EMR is easy to use | 3.22(1.05) | 1.11 | ** | 0.05 | 1.07 | ** | 0.06 |
|  | epd_16_t4 | | | Learning to use the EMR is easy for me | 3.71(0.93) | 0.79 | ** | 0.05 | 0.73 | ** | 0.05 |
|  |  | | | 1=completely disagree, 5=completely agree |  |  |  |  |  |  |  |
| **EMR aligned to dailyroutine**  (Cronbach’s alpha 0.92)  *Covaries with* | | | | Working with the EMR….. |  |  |  |  |  |  |  |
|  | epd_15_t1 | | | is well aligned with all aspects of my work | 3.44(0.96) | 1.00 |  |  | 1.00 |  |  |
| *epd_15_t3* | epd_15_t2 | | | suits the way I like to work | 3.51(1.05) | 1.05 | ** | 0.05 | 1.09 | ** | 0.05 |
| *epd_15_t2* | epd_15_t3 | | | suits my working style | 3.55(1.02) | 1.00 | ** | 0.05 | 0.98 | ** | 0.05 |
|  |  | | | 1=completely disagree, 5=completely agree |  |  |  |  |  |  |  |
| **Added value of EMR**  (Cronbach’s alpha 0.93) |  | | | Using the EMR..... |  |  |  |  |  |  |  |
|  | epd_14_t1 | | | enables me to do my tasks more quickly | 3.12(1.12) | 1.00 |  |  | 1.00 |  |  |
|  | epd_14_t2 | | | improves the quality of my work | 3.50(0.96) | 0.95 | ** | 0.05 | 0.86 | ** | 0.05 |
|  | epd_14_t3 | | | makes working easier | 3.41(1.02) | 1.02 | ** | 0.05 | 1.02 | ** | 0.05 |
|  | epd_14_t4 | | | increases my efficacy at work | 3.35(1.06) | 1.02 | ** | 0.05 | 1.11 | ** | 0.05 |
|  | epd_14_t5 | | | increases control over my work | 3.54(0.98) | 0.92 | ** | 0.05 | 0.90 | ** | 0.05 |
|  |  | | | 1=completely disagree, 5=completely agree |  |  |  |  |  |  |  |
|  |  | | |  |  |  |  |  |  |  |  |
| **Timeliness of use** | | | |  |  |  |  |  |  |  |  |
|  | epd_13 | | | When do you usually enter the patient data (e.g. treatment, medication) into the electronic medical file? | 2.03(0.97) | 1.00 |  |  | 1.00 |  |  |
|  |  | | | 6.Mostly during the patient visit; 5.Mostly after seeing one patient; 4.Mostly after seeing a number of patients. 3.Mostly at the end of my shift; 2.Mostly at the end of the week; 1.Mostly at the end of the month |  |  |  |  |  |  |  |
|  |  |  |  |  |  |  |  |  |  |  |  |
| **Quality of data**  (Cronbach’s alpha 0.70) | | |  | |  |  |  |  |  |  |  |
|  | epd_12_t1_R | | How often does it happen that you find data in patient files that do not match reality? | | 3.48(0.75) | 1.00 |  |  | 1.00 |  |  |
|  | epd_12_t2_R | | How often do you find patient data missing? | | 3.01(0.82) | 0.97 | ** | 0.10 | 1.26 | ** | 0.12 |
|  | epd_12_t3_R | | How often is it that you cannot enter the care provided into the patient file? | | 3.74(0.98) | 1.02 | ** | 0.11 | 1.05 | ** | 0.11 |
|  |  | | 5=never; 4=rarely; 3= sometimes; 2=regularly; 1=always | |  |  |  |  |  |  |  |
