# Supplementary material for: Use of electronic medical records and quality of patient data: different reaction patterns of doctors and nurses to the hospital organization
Source: BMC Med Inform Decis Mak. 2017 Feb 10;17:17. doi: 10.1186/s12911-017-0412-x (PMC5303309; doi:10.1186/s12911-017-0412-x)
Supplement: Additional file 3: — Codebook to the data. Variable names in the data and translated complete wording of items in questionnaire. Names of variables in Additional file 2 and the items in complete wording as presented in the Quesionnaire, translation from Dutch. (DOCX 19 kb) [file 12911_2017_412_MOESM3_ESM.docx]

Additional file 3 Variable names and items in the dataset

|  |  |  | |
| --- | --- | --- | --- |
|  |  |  | |
| Latent | variables: |  | |
| **Timeliness of use** | |  | |
|  | epd_13_clen_R | When do you usually enter the patient data (e.g. treatment, medication) into the electronic medical file? | |
|  |  | 5.Mostly during the patient visit; 4.Mostly after seeing one patient; 3.Mostly after seeing a number of patient Mostly after the end of my shift; 2.Mostly at the end of the week; 1.Mostly at the end of the month | |
| **Quality of data** | |  | |
|  | epd_12_t1_R | How often does it happen that you find data in patient files that do not match reality? | |
|  | epd_12_t2_R | How often do you miss patient data? | |
|  | epd_12_t3_R | How often does it happen that you cannot enter provided care into the patient file? | |
|  |  | 5=never; 4= in exceptional cases; 3= sometimes; 2=regularly; 1=always | |
|  | | |  |
|  | epd_16_t5_R | I will avoid working with my EMR whenever I can. | |
|  |  | 1=completely disagree, 5=completely agree | |
| **EMR easy to work with** | |  | |
|  | epd_16_t1 | Working with the EMR is clear and easy to understand | |
|  | epd_16_t2 | It is easy for me to make the EMR do what I want | |
|  | epd_16_t3 | The EMR is easy to use | |
|  | epd_16_t4 | Learning to use the EMR is easy for me | |
|  |  | 1=completely disagree, 5=completely agree | |
| **EMR aligned to daily routine** | | Working with the EMR….. | |
|  | epd_15_t1 | Is well aligned with all aspects of my work | |
|  | epd_15_t2 | suits the way I like to work | |
|  | epd_15_t3 | suits my working style | |
|  |  | 1=completely disagree, 5=completely agree | |
| Using the EMR | | |  |
|  | epd_14_t1 | Enables quicker realization of my tasks | |
|  | epd_14_t2 | improves the quality of my work | |
|  | epd_14_t3 | makes working easier | |
|  | epd_14_t4 | increases my efficacy at work | |
|  | epd_14_t5 | increases control over my work | |
|  |  | 1=completely disagree, 5=completely agree | |
| Management of this hospital…. | | |  |
|  | epd_18_t1 | engages employees to improve the interaction with others within the hospital | |
|  | epd_18_t2 | analyses relevant information before making a decision | |
|  | epd_18_t3 | lets me know how they judge my skills | |
|  | epd_18_t4 | admits mistakes | |
|  | epd_18_t5 | listens carefully to various points of view before drawing conclusions | |
|  | epd_18_t6 | knows when to reconsider her points of view | |
|  | epd_18_t7 | encourages everybody to express his or her opinion | |
|  | epd_18_t8 | supports the employees | |
|  |  | 1=completely disagree, 5=completely agree | |
| The administrative department….. | | |  |
|  | epd_24_t1 | reacts swiftly an adequately when there are problems in registration in the EMR | |
|  | epd_24_t2 | possess the necessary skills and expertise to support proper functioning of the EMR | |
|  | epd_24_t3 | possess the necessary skills and expertise to align the EMR to my work | |
|  |  | 1=completely disagree, 5=completely agree | |
| **IT support** | | The IT department….. | |
|  | epd_23_t1 | reacts swiftly an adequately when there are problems in registration in the EMR | |
|  | epd_23_t2 | possess the necessary skills and expertise to support proper functioning of the EMR | |
|  | epd_23_t3 | possess the necessary skills and expertise to align the EMR to my work | |
|  |  | 1=completely disagree, 5=completely agree | |
| **HR support** | | The HR department….. | |
|  | epd_22_t1 | reacts swiftly an adequately when there are problems in registration in the EMR | |
|  | epd_22_t2 | possess the necessary skills and expertise to support proper functioning of the EMR | |
|  | epd_22_t3 | possess the necessary skills and expertise to align the EMR to my work | |
|  |  | 1=completely disagree, 5=completely agree | |
| Bottom up implementation | | |  |
|  | epd_21_t1 | I was asked on how the EMR should be implemented beforehand | |
|  | epd_21_t2 | Prior to the implementation, I was asked what I needed to do my work better | |
|  | epd_21_t3 | During implementation I was able to tell what I thought of the implementation | |
|  | epd_21_t4 | During implementation I was able to tell what I wanted different in the EMR | |
|  |  | 1=completely disagree, 5=completely agree | |
| **Open culture** | |  | |
|  | epd_29_t1 | If I feel that I did not do my job as I should, I will discuss this with my team members | |
|  | epd_29_t2 | Regularly I ask my team members for advice | |
|  | epd_29_t3 | If I feel that I did not do my job as I should, I will discuss this with my manager | |
|  | epd_29_t4 | Regularly I ask my manager for advice | |
|  |  | 1=completely disagree, 5=completely agree | |
|  | | |  |
|  | epd_27_t1 | My hospital encourages me to try new ideas that may lead to improved work processes | |
|  | epd_27_t2 | If I try to improve a work process, my colleagues are open to it | |
|  | epd_27_t4 | My manager will listen if I suggest an idea to improve the work proces | |
|  |  | 1=completely disagree, 5=completely agree | |
|  |  |  | |
|  | vplk | Dummy nurse (1=nurse, 0=doctor) | |
|  | iplement | Implementation stage 1-3. Higher -> further stage of implementation | |
