# Supplementary material for: Use of electronic medical records and quality of patient data: different reaction patterns of doctors and nurses to the hospital organization
Source: BMC Med Inform Decis Mak. 2017 Feb 10;17:17. doi: 10.1186/s12911-017-0412-x (PMC5303309; doi:10.1186/s12911-017-0412-x)
Supplement: Additional file 4: — Complete Dutch questionnaire. (DOCX 25 kb) [file 12911_2017_412_MOESM4_ESM.docx]

**{avars_intro}**

Allereerst vragen we enkele van uw persoonskenmerken. Deze gegevens worden gebruikt om de representativiteit van het onderzoek te verifiëren. We willen hiermee controleren of we voldoende mannen en vrouwen hebben in de steekproef, of we alle leeftijdsgroepen hebben, en of we alle functies hebben die we nodig hebben.

**avars_geslacht**

U bent:

1 Man

2 Vrouw

**avars_gebjaar**

Uw geboortejaar:

*1900..2010 (dropdown)*

**avars_ziekenhuis**

In wat voor soort ziekenhuis werkt u?

1 Academisch

2 Top klinisch

3 Algemeen

4 Categoraal

5 Zelfstandig Behandelcentrum

6 Anders, namelijk....

*if avars_ziekenhuis=6*

**avars_ziekenhuis_and**

*string*

**avars_arbeidswerkduur**

Hoe lang werkt u al voor uw huidige ziekenhuis? (in jaren)

*0..100* jaar

**avars_afdeling**

Op welke afdeling werkt u?

*string*

**avars_functieduur**

Hoe lang werkt u al in uw huidige functie? (in jaren)

*0..100* jaar

**avars_beroep**

Waar valt uw beroep/functie onder?

1 Specialist

2 Arts in opleiding tot specialist (AIOS)

3 Arts-assistent niet in opleiding tot specialist (ANIOS)

4 Hoofdverpleegkundige

5 Verpleegkundige

6 Nurse practitioner

7 Verpleegkundig specialist

8 Physician assistant

9 Paramedicus

10 Anders, namelijk

*if avars_beroep=10*

**avars_beroep_and**

*string*

**{intro2}**

**Mogelijkheden bij het elektronische patiëntendossier**

De volgende vragen zijn een inventarisatie van de mogelijkheden van de statusvoering in uw elektronisch patiëntendossier. In sommige ziekenhuizen wordt het patiëntendossier geheel elektronisch gedaan, in andere is het een mix van elektronisch en papier.

**epd_01**

Wordt het patiëntendossier in uw ziekenhuis geheel elektronisch gedaan of deels op papier en deels elektronisch?

1 Geheel elektronisch

2 Deels op papier, deels elektronisch

*if (epd_01 = 1)*

**epd_03**

Bevindt het hele patiëntendossier zich in één systeem?

1 Ja

2 Nee

*if (epd_03 = 2)*

**epd_04**

Ongeveer hoeveel systemen moet u maximaal gebruiken bij de statusvoering van patiënten?

*0..100*

**epd_09_t1 - epd_09_t3**

**epd_09_t1** Geeft het EPD u advies over behandelmogelijkheden per patiënt?

**epd_09_t2** Is de statusvoering van artsen in te zien door verpleegkundigen?

**epd_09_t3** Is de statusvoering van verpleegkundigen in te zien door artsen?

1 Ja

2 Nee

*if (avars_beroep=2) or (avars_beroep=3)*

**epd_10t1 - epd_10t2**

Zoals u weet is de DBC systematiek 1 januari 2012 van dit jaar overgegaan op de DOT systematiek. De registratie van diagnose en verrichtingen leiden tot een DOT zorgproduct. Dat zorgproduct (uw geleverde zorg) wordt afgeleid uit uw registratie door een landelijke systeem, de Grouper. Onjuiste, onvolledige en niet tijdige registratie kunnen tot een verkeerd of geen zorgproduct leiden waardoor te veel/te weinig of niet kan worden gedeclareerd.

**In de volgende vragen kunt u aangeven in hoeverre de registratie aansluit bij de zorgpraktijk.**

**epd_10t1** Ik leg een verwacht zorgproduct vast (eventueel in stappen of dmv keuzemenu's)

**epd_10t2** Ik kan nog steeds een DBC vastleggen (eventueel in stappen of dmv keuzemenu's)

1 Ja

2 Nee

3 n.v.t.

4 Ik ken het begrip niet

*if (avars_beroep=2) or (avars_beroep=3)*

**epd_10t3 - epd_11_t2**

**epd_10t3** Controleert het systeem of de geregistreerde diagnose in combinatie met de verrichtingen passend is?

**epd_10t4** Kunt u achteraf de diagnose wijzigen als u later bedenkt dat het toch niet klopte wat u eerder had ingevoerd?

**epd_11_t1** Hebt u voorafgaand aan de invoering van DOT uitleg gehad over de veranderingen in de registratie?

**epd_11_t2** Hebt u voorafgaand aan de invoering van DOT een cursus of training gekregen?

1 Ja

2 Nee

**epd_12_t1 - epd_12_t5**

Bij het registreren van activiteiten in systemen kan het voorkomen dat u in meer of mindere mate afwijkt van de daadwerkelijke behandeling, omdat het systeem niet de ruimte biedt de daadwerkelijk verleende zorg te registreren.

**epd_12_t1** Hoe vaak komt het voor dat u gegevens in een patiëntendossier aantreft die niet blijken te kloppen?

**epd_12_t2** Hoe vaak komt het voor dat gegevens ontbreken?

**epd_12_t3** Hoe vaak komt het voor dat u de feitelijk verleende zorg niet kunt registreren in het patiëntendossier?

**epd_12_t4** Hoe vaak vergeet u zorghandelingen (verrichtingen) te registreren in het patiëntendossier?

**epd_12_t5** Hoe vaak komt het voor dat het EPD geen mogelijkheid bevat voor patiëntinformatie die u nodig heeft voor behandeling?

*(bijvoorbeeld overzicht actuele medicatie buiten ziekenhuis of informatie van huisarts)*

1 Nooit

2 Bij uitzondering

3 Soms

4 Regelmatig

5 Altijd

**epd_12_t5_open**

Als u "regelmatig" of "altijd" heeft ingevuld, welke informatie zit niet structureel in uw EPD die u wel regelmatig nodig heeft?

*string, empty*

**epd_12_open**

Wilt u iets toevoegen aan bovenstaande antwoorden?

*(U kunt deze vraag ook leeg laten)*

*open, empty*

*if (avars_beroep <= 8)*

**epd_13**

**Tijdigheid**

Wanneer registreert u (gewoonlijk) de noodzakelijke gegevens (bijvoorbeeld behandeling, toegediende medicatie) in het elektronische patiëntendossier?

1 Meestal tijdens het zien van de patiënt

2 Meestal direct nadat ik één patiënt heb gezien

3 Meestal nadat ik een aantal patiënten heb gezien

4 Meestal aan het eind van de dienst

5 Meestal aan het eind van de week

6 Meestal aan het eind van de maand

7 Anders, namelijk...

*if (epd_13 = 7)*

**epd_13_and**

*string*

**{intro3}**

**Ervaringen en mening over het elektronische patiëntendossier (EPD)**

De volgende vragen gaan over uw ervaringen bij het gebruik van het patiëntendossier. Het gaat om het elektronische deel van het patiëntendossier. Indien u een deel op papier doet en een deel elektronisch, wilt u dan antwoorden voor het elektronische deel van het dossier?

**epd_14_t1 - epd_14_t5**

**Het gebruik van het EPD ...**

**epd_14_t1** ... maakt het mogelijk dat ik mijn taken sneller uitvoer.

**epd_14_t2** ... verbetert de kwaliteit van het werk dat ik doe.

**epd_14_t3** ... maakt het werken makkelijker.

**epd_14_t4** ... vergroot mijn effectiviteit op het werk.

**epd_14_t5** ... geeft me meer controle over mijn werk.

1 Helemaal oneens

2 Eerder oneens

3 Niet eens, niet oneens

4 Eerder eens

5 Helemaal eens

**epd_15_t1 - epd_15_t3**

**Werken met het EPD ...**

**epd_15_t1** ... sluit goed aan bij alle aspecten van mijn werk.

**epd_15_t2** ... past goed bij de manier waarop ik graag werk.

**epd_15_t3** ... past bij mijn werkstijl.

1 Helemaal oneens

2 Eerder oneens

3 Niet eens, niet oneens

4 Eerder eens

5 Helemaal eens

**epd_16_t1 - epd_16_t7**

**In hoeverre bent u het eens of oneens met de volgende uitspraken?**

**epd_16_t1** Het werken met het EPD is duidelijk en begrijpelijk.

**epd_16_t2** Het is makkelijk om het EPD te laten doen wat ik wil dat het doet.

**epd_16_t3** Het EPD is makkelijk in het gebruik.

**epd_16_t4** Het leren gebruiken van het EPD is makkelijk voor mij.

**epd_16_t5** Ik vermijd het werken met het EPD wanneer dit enigszins mogelijk is.

**epd_16_t6** Het werken met het EPD verbetert de kwaliteit van de zorg.

**epd_16_t7** Het werken met het EPD verbetert de patiëntveiligheid.

1 Helemaal oneens

2 Eerder oneens

3 Niet eens, niet oneens

4 Eerder eens

5 Helemaal eens

**epd_16_open**

Wilt u verder nog iets kwijt over het werken met het elektronische patiëntendossier? *(U kunt deze vraag ook leeg laten)*

*open, empty*

**epd_17_t5 -** **epd_17_t6**

De volgende vragen gaan over de wijze waarop het ziekenhuismanagement de invoer van het EPD begeleid. In welke mate bent u het eens of oneens met de volgende uitspraken?

**epd_17_t5** Ik ben in staat om mijn dagelijkse werkzaamheden zonder hinder voort te zetten terwijl het EPD wordt *ingevoerd*.

**epd_17_t6** Ik ben in staat om mijn dagelijkse werkzaamheden zonder hinder voort te zetten terwijl het EPD wordt *uitgebreid*.

1 Helemaal oneens

2 Eerder oneens

3 Niet eens, niet oneens

4 Eerder eens

5 Helemaal eens

**epd_17_open**

Wilt u nog iets kwijt over de wijze waarop het ziekenhuismanagement de invoer van het EPD begeleid?

*(U kunt deze vraag ook leeg laten)*

*open, empty*

**epd_18_t1 - epd_18_t8**

Hieronder vragen we wat u vindt van het algemene optreden van het ziekenhuismanagement.

**Het management van dit ziekenhuis...**

**epd_18_t1** ...gaat het gesprek aan om de interactie met anderen binnen het ziekenhuis te verbeteren.

**epd_18_t2** ...analyseert relevante gegevens alvorens een beslissing te nemen.

**epd_18_t3** ...laat weten hoe ze over mijn bekwaamheden denkt.

**epd_18_t4** ...geeft gemaakte fouten toe.

**epd_18_t5** ...luistert zorgvuldig naar de verschillende standpunten voordat ze een conclusie trekt.

**epd_18_t6** ...weet wanneer het tijd is om haar standpunten opnieuw te bekijken.

**epd_18_t7** ...spoort iedereen aan om voor zijn of haar mening uit te komen.

**epd_18_t8** ...staat achter de medewerkers.

1 Helemaal oneens

2 Eerder oneens

3 Niet eens, niet oneens

4 Eerder eens

5 Helemaal eens

**epd_19_t1 - epd_19_t9**

De volgende uitspraken gaan over de wijze waarop in uw ziekenhuis wordt omgegaan met technologische vernieuwingen, zoals het EPD. In welke mate bent u het hiermee eens of oneens?

**Bij het doorvoeren van een technologische vernieuwing...**

**epd_19_t1** ...is vooraf duidelijk wat de reden voor de vernieuwing is.

**epd_19_t2** ...zijn vooraf succesfactoren benoemd.

**epd_19_t3** ...wordt het traject gefaseerd ingevoerd.

**epd_19_t4** ...is duidelijk wie verantwoordelijk is voor het succes ervan.

**epd_19_t5** ...wordt ervoor gezorgd dat het personeel over de benodigde kennis beschikt.

**epd_19_t6** ...worden vooraf meetbare doelen vastgesteld.

**epd_19_t7** ...vindt tussentijdse evaluatie plaats.

**epd_19_t8** ...hebben veel mensen persoonlijk belang bij het slagen ervan.

**epd_19_t9** ...wordt het stoppen ervan als een afgang gezien.

1 Helemaal oneens

2 Eerder oneens

3 Niet eens, niet oneens

4 Eerder eens

5 Helemaal eens

**epd_20_t1 - epd_20_t2**

**In dit ziekenhuis...**

**epd_20_t1** ...wordt een standaard werkwijze gebruikt bij implementaties.

**epd_20_t2** ...wordt er geleerd van fouten in het verleden.

1 Helemaal oneens

2 Eerder oneens

3 Niet eens, niet oneens

4 Eerder eens

5 Helemaal eens

**epd_21_t1 - epd_21_t4**

**Verspreidingsproces:**

**epd_21_t1** Vooraf is mij gevraagd op welke wijze het EPD zou moeten worden ingevoerd.

**epd_21_t2** Vooraf aan de implementatie van het EPD is me gevraagd wat ik nodig zou hebben om mijn werk beter te doen.

**epd_21_t3** Tijdens de uitrol heb ik kunnen vertellen wat ik vond van de wijze van implementatie.

**epd_21_t4** Tijdens de uitrol heb ik kunnen vertellen wat ik anders wilde in het EPD.

1 Helemaal oneens

2 Eerder oneens

3 Niet eens, niet oneens

4 Eerder eens

5 Helemaal eens

**epd_21_open**

Wilt u verder nog iets kwijt over de wijze waarop het EPD in uw ziekenhuis wordt ingevoerd?

*(U kunt deze vraag ook leeg laten)*

*open, empty*

**epd_22_t1 - epd_22_t3**

De volgende uitspraken gaan over de wijze waarop verschillende afdelingen de uitrol van het EPD ondersteunen en begeleiden. In welke mate bent u het hiermee eens of oneens?

**De personeelsafdeling (HR of P&O)...**

**epd_22_t1** ...biedt trainingen en cursussen aan wanneer er wijzigingen plaatsvinden in ons EPD.

**epd_22_t2** ...streeft er naar om de trainingen en cursussen aan te laten sluiten op onze bijscholingsvragen die volgen uit het werken met het EPD.

**epd_22_t3**...heeft voldoende tijd en middelen om voor ons de trainingen en cursussen aan te bieden die aansluiten op onze bijscholingsvragen die volgen uit het werken met het EPD.

1 Helemaal oneens

2 Eerder oneens

3 Niet eens, niet oneens

4 Eerder eens

5 Helemaal eens

**epd_23_t1 - epd_23_t3**

**De ICT afdeling...**

**epd_23_t1** ...reageert snel en adequaat bij problemen rond de registratie van het EPD.

**epd_23_t2** ...heeft de nodige kennis en expertise om het EPD goed te laten werken.

**epd_23_t3** ...heeft de nodige kennis en expertise om het EPD aan te laten sluiten op mijn werk.

1 Helemaal oneens

2 Eerder oneens

3 Niet eens, niet oneens

4 Eerder eens

5 Helemaal eens

**epd_24_t1 - epd_24_t3**

**De afdeling zorgadministratie/DBC helpdesk...**

**epd_24_t1** ...reageert snel en adequaat bij problemen rond de registratie van het EPD.

**epd_24_t2** ...heeft de nodige kennis en expertise om het EPD goed te laten werken.

**epd_24_t3** ...heeft de nodige kennis en expertise om het EPD aan te laten sluiten op mijn werk.

1 Helemaal oneens

2 Eerder oneens

3 Niet eens, niet oneens

4 Eerder eens

5 Helemaal eens

**epd_24_open**

Wilt u verder nog iets kwijt over het functioneren van de verschillende afdelingen bij de invoering van het EPD?

*(U kunt deze vraag ook leeg laten)*

*open, empty*

**epd_leiding**

Hebt u een leidinggevende?

1 Ja

2 Nee

*if epd_leiding=1*

**{epd_25_intro}**

De leidinggevende vertaalt het beleid van de organisatie naar de praktijk. Ook hij/zij kan een belangrijke rol spelen bij de invoering van een nieuw systeem. De volgende vragen gaan daarom over uw leidinggevende. We benadrukken nogmaals dat uw antwoorden niet herleidbaar zijn.

*if epd_leiding=1*

**epd_25**

**Mijn leidinggevende is:**

1 Specialist

2 Hoofdverpleegkundige

3 Manager

4 Afdelingshoofd (eventueel specialist)

5 Anders, namelijk...

*if (epd_25=5)*

**epd_25_and**

*string*

*if epd_leiding=1*

**epd_26_t1 - epd_26_t6**

**Mijn leidinggevende...**

**epd_26_t1** zorgt ervoor dat het beleid van het managementteam wordt omgezet in concrete veranderingen.

**epd_26_t2** heeft oog voor de problemen van de medewerkers wanneer er iets wordt veranderd bij het EPD.

**epd_26_t3** zorgt ervoor dat onze wensen het EPD worden overgebracht.

**epd_26_t4** krijgt vanuit de organisatie voldoende mensen en middelen voor het uitvoeren van de veranderingen betreffende het EPD.

**epd_26_t5** wordt gesteund door het hogere management.

**epd_26_t6** staat achter zijn/haar mensen.

1 Helemaal oneens

2 Eerder oneens

3 Niet eens, niet oneens

4 Eerder eens

5 Helemaal eens

**epd_27_t1 - epd_27_t4**

Nu volgen wat vragen over de cultuur van uw ziekenhuis en uw ervaring met patiëntendossiers.

**Cultuur**

De cultuur van een bedrijf bestaat uit veel dimensies en komt o.a. tot uiting in wat mensen gebruikelijk vinden in bepaalde situaties. Met onderstaande vragen willen we achterhalen wat u vindt dat gebruikelijk is bij veranderingen in uw ziekenhuis.

**In welke mate komen de volgende situaties voor?**

**epd_27_t1** In mijn ziekenhuis word je aangemoedigd om ideeën uit te proberen die kunnen leiden tot betere werkprocessen.

**epd_27_t2** Als ik een werkproces probeer te verbeteren, staan mijn collega's daar voor open.

**epd_27_t3** Als ik een werkproces probeer te verbeteren en het blijkt niet te werken, zullen mijn collega's daar over roddelen.

*if epd_leiding=1*

**epd_27_t4** Mijn leidinggevende zal luisteren als ik hem/haar een idee voorleg voor verbetering van een werkproces.

1 Nooit

2 Bijna nooit

3 Soms

4 Vaak

5 Altijd

**epd_28_t1 - epd_28_t5**

**Persoonlijke leerstijl en werkstijl**

Iedere persoon heeft een eigen leer- en werkstijl. Sommige organisatorische aspecten sluiten beter aan bij de ene werkstijl dan bij de andere. Daarom enkele vragen over de manier waarop u (het liefste) werkt.

Er zijn geen goede of foute antwoorden, het gaat in alle gevallen over uw persoonlijke stijl.

**In welke mate bent u het eens of oneens met de volgende uitspraken?**

**epd_28_t1** Ik geef de voorkeur aan gestructureerd werk.

**epd_28_t2** Ik vind het fijn wanneer in de organisatie functie-eisen en werkinstructies duidelijk en gedetailleerd uitgelegd zijn zodat iedereen altijd precies weet wat ze moeten doen.

**epd_28_t3** Ik geef de voorkeur aan werk waarbij werkinstructies in detail zijn uitgeschreven.

**epd_28_t4** Ik vind het fijn om voor leidinggevenden te werken die verwachten dat werknemers altijd nauw instructies en procedures opvolgen.

**epd_28_t5** Regels en procedures zijn belangrijk omdat iedereen dan weet wat er van hem verwacht wordt.

1 Helemaal oneens

2 Eerder oneens

3 Niet eens, niet oneens

4 Eerder eens

5 Helemaal eens

**epd_29_t1 - epd_29_t4**

**In welke mate komen de volgende situaties voor?**

**epd_29_t1** Als ik vind dat ik mijn werk niet goed heb gedaan dan bespreek ik dit met mijn teamleden.

**epd_29_t2** Ik vraag mijn teamleden regelmatig om advies.

*if epd_leiding=1*

**epd_29_t3** Als ik vind dat ik mijn werk niet goed heb gedaan dan bespreek ik dit met mijn leidinggevende.

*if epd_leiding=1*

**epd_29_t4** Ik vraag mijn leidinggevende regelmatig om advies.

1 Nooit

2 Bijna nooit

3 Soms

4 Vaak

5 Altijd

**epd_30_t1 - epd_30_t7**

**In welke mate bent u het eens of oneens met de volgende uitspraken?**

**epd_30_t1** Ik kom met creatieve oplossingen voor problemen.

**epd_30_t2** Ik ga op zoek naar nieuwe werkvormen die effectiever zijn.

**epd_30_t3** Ik promoot en verdedig mijn innovatieve ideeën bij anderen.

**epd_30_t4** Ik probeer mijn teamleden te overtuigen van een innovatieve werkwijze.

**epd_30_t5** Ik toon creativiteit in mijn werk wanneer ik daar de mogelijkheid voor krijg.

**epd_30_t6** Ik probeer overeenstemming te bereiken over nieuwe manieren voor het uitvoeren van taken.

**epd_30_t7** Ik probeer zelf nieuwe werkprocessen uit.

1 Helemaal oneens

2 Eerder oneens

3 Niet eens, niet oneens

4 Eerder eens

5 Helemaal eens

**opm**

Tot slot. Hebt u nog opmerkingen over deze vragenlijst?

*Klikt u op Verder totdat u het bericht krijgt dat u het venster kunt sluiten. Pas dan registreert het systeem de vragenlijst als* ***volledig*** *ingevuld*.

1 Ja

2 Nee

*if (opm=1)*

**evaopm**

U kunt uw opmerking hieronder invullen.

*open*
